# Senior-COVID-Rea Cohort Study: A Geriatric Prediction Model of 30-day Mortality in Patients Aged over 60 Years in ICU for Severe COVID-19

**DOI:** 10.14336/AD.2021.1004

**Published:** 2022-04-01

**Authors:** Claire Falandry, Laurent Bitker, Paul Abraham, Fabien Subtil, Vincent Collange, Baptiste Balança, Max Haïne, Céline Guichon, Christophe Leroy, Marie Simon, Amélie Malapert, Jean-Baptiste Pialat, Laurent Jallades, Alain Lepape, Arnaud Friggeri, Fabrice Thiolliere

**Affiliations:** ^1^Hospices Civils de Lyon, Service de Gériatrie, Centre Hospitalier Lyon Sud, Pierre-Bénite, France.; ^2^Université de Lyon, Laboratoire CarMeN, Inserm U1060, INRA U1397, Université Claude Bernard Lyon 1, INSA Lyon, Charles Mérieux Medical School, Pierre-Bénite, France.; ^3^Hospices Civils de Lyon, Service de Réanimation Médicale, Hôpital de La Croix Rousse, Lyon, France.; ^4^Université de Lyon, CREATIS INSERM 1044 CNRS 5220, Villeurbanne, France.; ^5^Hospices Civils de Lyon, Département d’anesthésie et reanimation médicale, Hôpital Edouard Herriot, Lyon, France.; ^6^Université de Lyon, CNRS, UMR5558, Laboratoire de Biométrie et Biologie Evolutive, Université Claude Bernard Lyon 1, Villeurbanne, France.; ^7^Hospices Civils de Lyon, Service de Biostatistique, Lyon, France.; ^8^Medipole Lyon-Villeurbanne, Département anesthésie réanimation, Villeurbanne, France.; ^9^Hospices Civils de Lyon, Département of d’Anesthésie Réanimation Neurologique, Hôpital Wertheimer, Bron, France.; ^10^Hôpital Pierre Wertheimer; Université de Lyon, Inserm U1028, CNRS UMR 5292, Lyon Neuroscience Research Centre, Team TIGER, Bron, France.; ^11^Hôpital Nord-Ouest, Service de Gériatrie, Gleizé, France.; ^12^Hospices Civils de Lyon, Service de réanimation, Centre hospitalier universitaire de la Croix Rousse, Lyon, France.; ^13^Université de Lyon, Lyon, France.; ^14^Centre Hospitalier Emile Roux, Service de réanimation, Le Puy-en-Velay, France.; ^15^Hospices Civils de Lyon, Service de Médecine Intensive-Réanimation Médicale, Hôpital Edouard Herriot, Lyon, France.; ^16^Hospices Civils de Lyon, Plateforme Transversale de Recherche de l'ICHCL, Pierre-Bénite, France.; ^17^Hospices Civils de Lyon, Service de radiologie, Groupement Hospitalier Sud; Pierre-Bénite, France.; ^18^Université de Lyon, CREATIS CNRS UMR 5220 INSERM U1206, Villeurbanne, France.; ^19^Hospices Civils de Lyon, Service d'Hématologie biologique - Groupement Hospitalier Sud, Pierre-Bénite, France.; ^20^Hospices Civils de Lyon, Service d’Anesthésie-Réanimation, Centre Hospitalier Lyon Sud, Pierre-Bénite, France.; ^21^Université de Lyon, Centre International de Recherche en Infectiologie (CIRI), Lyon, France. On behalf of the Senior-COVID-Rea study group. ^Membership of the Senior-COVID-Rea study group is provided in the Acknowledgments section.;

**Keywords:** triage, intensive care, geriatric parameters, COVID-19

## Abstract

The SARS-COV2 pandemic induces tensions on health systems and ethical dilemmas. Practitioners need help tools to define patients not candidate for ICU admission. A multicentre observational study was performed to evaluate the impact of age and geriatric parameters on 30-day mortality in patients aged ≥60 years of age. Patients or next of kin were asked to answer a phone questionnaire assessing geriatric covariates 1 month before ICU admission. Among 290 screened patients, 231 were included between March 7 and May 7, 2020. In univariate, factors associated with lower 30-day survival were: age (per 10 years increase; OR 3.43, [95%CI: 2.13-5.53]), ≥3 CIRS-G grade ≥2 comorbidities (OR 2.49 [95%CI: 1.36-4.56]), impaired ADL, (OR 4.86 [95%CI: 2.44-9.72]), impaired IADL8 (OR 6.33 [95%CI: 3.31-12.10], p<0.001), frailty according to the Fried score (OR 4.33 [95%CI: 2.03-9.24]) or the CFS ≥5 (OR 3.79 [95%CI: 1.76-8.15]), 6-month fall history (OR 3.46 [95%CI: 1.58-7.63]). The final multivariate model included age (per 10 years increase; 2.94 [95%CI:1.78-5.04], p<0.001) and impaired IADL8 (OR 5.69 [95%CI: 2.90-11.47], p<0.001)). Considered as continuous variables, the model led to an AUC of 0.78 [95% CI: 0.72, 0.85]. Age and IADL8 provide independent prognostic factors for 30-day mortality in the considered population. Considering a risk of death exceeding 80% (82.6% [95%CI: 61.2% - 95.0%]), patients aged over 80 years with at least 1 IADL impairment appear as poor candidates for ICU admission.

At the end of December 2019, the severe acute respiratory syndrome coronavirus 2 (SARSCoV-2) and the disease it causes, coronavirus disease 2019 (COVID-19), became rapidly an emerging pandemic and a public health challenge [1]. The older population was rapidly identified as having the highest risk for severe complications including acute respiratory distress syndrome (67-71%), acute kidney injury (20-29%), acute cardiac injury (23-33%) or liver dysfunction (15-29%), and a 5-fold increased risk for premature death [1-3]; this leads to a frequent theoretical indication for intensive care unit (ICU) transfer in this population. However, the first published data suggested a low benefit of ICU admission for patients aged over 70 years, and that this was almost absent in patients aged over 80 years [2, 4, 5], but this does not take into account the heterogeneity of this population [6] and therefore the risk/benefit balance of an ICU transfer may be questioned at the individual level, independently of care resources.

Cardiovascular comorbidities [7], laboratory covariates [8, 9], the time since the first symptoms [5], and ICU-specific scores [5] have been found to be associated with ICU mortality in COVID-19 patients. The identification of such variables led to the development of prognostic models; these were compiled in a systematic review by Wynants et al. and had AUC values ranging from 0.68 to 0.99, however the authors highlighted the low quality of the published studies [10] and appealed for a broader use of Transparent Reporting of a multivariable prediction model for Individual Prognosis or Diagnosis (TRIPOD) reporting guidelines [11].

Among the available geriatric parameters, frailty is frequently used to explain the heterogeneity of the older population; it is defined as a state of increased vulnerability to poor resolution of homoeostasis after a stressor event, which increases the risk of adverse outcomes [12]. Some consensus has been reached on its definition, but there are two conceptual views as to its operational criteria: a multidomain view of frailty [13], and a phenotypical view of frailty, linked to malnutrition and sarcopenia [14]. In an even more pragmatic view of frailty, the Clinical Frailty Score (CFS) was developed; this stratifies older patients in to distinct levels of fitness according a rapid “at a glance” assessment based on the diagnosis of a specifically trained geriatrician [15]. Interestingly, according to recent evidence from 2 studies, disease outcomes of COVID-19 patients admitted to hospital would be better predicted by frailty - using different multidomain models of frailty assessments - than either age or comorbidity [16, 17]. In parallel, on March 20, 2020, the United Kingdom’s National Institute for Health and Care Excellence (NICE) published a COVID-19 rapid guideline; this indicated that only patients with a CFS <5 should be considered for critical care [18], as the threshold of 5 was previously shown to predict a higher mortality in (non-COVID) older patients admitted to ICU [19]. This guideline applies to those with the indication for ICU admission, and therefore should be considered as a non-admission decision help tool as opposed as an admission decision help tool. The geriatric community promptly reacted to these guidelines, pointing out the risk of drift in assessing the CFS, designed to be performed by trained geriatricians [20, 21], and the ethical dilemma of transforming the frailty spectrum into a binary covariate, considering that the inter-rater variability may be high between CFS scores 4 and 5 [21]. Moreover, two studies evaluating the impact of CFS score in COVID-19 versus non-COVID-19 populations found that CFS was not a good discriminator of prognosis in COVID-19 populations [22, 23]. There is therefore still a lack of non-admission decision help tools that may be applied for such patients. In line with that proposed by Christian et al. to deal with mass casualty events such as flu epidemics, exclusion criteria should be defined to identify patients who are not candidates for ICU admission including those: with a poor prognosis despite care in an ICU, requiring resources that cannot be provided, whose underlying illness has a poor prognosis with a high likelihood of death, and who are “too well” [24].

To better define the individual risk/benefit ratio of ICU admission, we conducted a multicentre observational study to determine the covariates predictive of mortality in the population of patients aged ≥60 years admitted to ICU, with a specific attention paid to their geriatric parameters 1 month before ICU admission.

## MATERIALS AND METHODS

The study protocol was extensively described elsewhere [25].

### Objectives

The primary objective was to evaluate the impact of age on 30-day mortality after ICU admission. A secondary objective was to construct a prognostic model for 30-day mortality based on co-morbidities, functional status of the patient 1 month before COVID-19 infection, laboratory data [8, 9, 26], radiological data [27], ICU parameters, and time since the first symptoms [25].

This analysis focused on the prognostic and discriminatory performance of the following geriatric covariates: number of grade ≥2 comorbidities according to the cumulative illness rating scale-geriatric (CIRS-G), activities of daily living (ADL) score [28], instrumental ADL, 8 variables (IADL8) score [29], Fried score, CFS, 6-month fall history. The CFS score ranges from 1 to 9; 1: very fit, 2: well, 3: managing well, 4: vulnerable, 5: mildly frail, 6: moderately frail, 7: severely frail, 8: very severely frail, 9: terminally ill.

### Study design

The Senior-COVID-Rea study was a multicentre observational cohort study. The study protocol (V1.0 of April 7, 2020) was approved by the ethics committee of the Hospices Civils de Lyon and declared on the ClinicalTrials platform (NCT04422340). According to the patient’s clinical condition, non-opposition was collected from the patient or next of kin if this was not possible, in accordance with the International Council for Harmonisation (ICH) Harmonised Guideline For Good Clinical Practice. The study follows the Strengthening the Reporting of Observational Studies in Epidemiology (STROBE) statement for the reporting of cohort studies [30] (Supplementary Table 1) and the TRIPOD guidelines for the reporting of prediction models [11] (Supplementary Table 2).

### Participants

All patients aged ≥60 admitted to the participating ICUs with a diagnosis of COVID-19 were screened centrally using computerized medical records. Diagnostic criteria were laboratory-confirmed SARS-CoV-2-positive swabs and/or a radiological diagnosis made by lung computed tomography (CT)-scan. Patients were included when non-opposition was collected. Patients who were transferred from one ICU to another were identified and their stay was considered as a single ICU admission. Screening logs of eligible participants were retained at each site.

After inclusion, a telephone-administered questionnaire explored the functional status of the patient 1 month before ICU admission. CFS assessment was either routinely fulfilled by the physicians in charge during the course of patient care or by a geriatrician based on the analysis of the medical and functional charts.

### Data collection

Data were collected across 7 ICUs in the Auvergne-Rhone-Alpes Region, France, 4 in referral university hospitals and 3 in primary care hospitals. Due to territorial collaborations, patients could be transferred from one ICU to the other during their stay and duplicate were deleted. A standardized case report form was used to collect data, including: comorbidities 1 month prior to infection (CIRS-G grade ≥2 [31]) and more specifically cardiac and vascular comorbidities (CIRS-G grade ≥2); the functional status 1 month before infection, assessed by the caregiver using the CFS and the ADL and IADL8 and 4 scores; nutritional data (weight at hospital and ICU admission, weight loss in the month and 6 months before infection, presence of mild or severe anorexia); laboratory data at ICU admission (LDH: lactate dehydrogenase, CRP: C reactive protein, and creatinine levels, as well as lymphocyte and neutrophil counts, and Sysmex haematological analyzer data [immature granulocyte count: IG;: high fluorescent lymphocyte count: HFLC; Sysmex, Kobe, Japan] (6-8)); chest imaging data (COVID-19 lung extension rated as minimal, moderate, extensive, severe, or critical according to the French Radiology Society guidelines [27]); and resuscitation parameters at ICU admission (arterial oxygen pressure/fraction of inspired oxygen [PaO2/FiO2] ratio, *indice de gravité simplifié II* - simplified acute physiology score II [IGS II-SASP II] [32]) and/or sepsis-related organ failure assessment (SOFA) score (*a posteriori* estimate based on IGS II-SASP II [33]), and interval between the first signs of infection and admission to ICU.

### Outcomes

The primary outcome was the 30-day mortality after ICU admission. No secondary outcome was considered herein.

### Statistical analysis

The first hypothesis of Senior-COVID-Rea was based on the first Chinese retrospective results [2]: considering a single analysis variable (age), with expected mortality of 30% in patients <70 years of age, and 70% in patients ≥70 years of age (with 40% of patients ≥70 years of age), a total of 130 patients was expected to show a significant difference with a power of 90% (bilateral alpha risk of 5%). Since the model of survival proposed in Senior-COVID-Rea analysis considered the integration of a maximum of 15 factors, aiming at a R^2^ of 0.5, and to achieve an optimism (the fact that the model accuracy is overestimated when it is assessed on the same sample as the one used to build the model) of ≤10%, 185 patients were to be included (criterion 1 of Riley and Snell [34]). After the publication of data on mortality in ICU in the Lombardy region of Italy [4], considering a risk of insufficient statistical power and selection bias, the study scientific committee decided that all the patients admitted to ICU before the May 7, 2020 should be screened and invited to participate in the study. This sample size calculation was modified on Clinicaltrials.gov site accordingly (July 28, 2020).

Continuous variables were described by the mean, standard deviation (SD), and range. Categorical variables were described by the frequency and percentage for each level. Commonly used thresholds were applied: CFS ≥5 [35], ADL <6 [28], IADL8 <8 [29], Fried score >2 [14].

The effect of factors on day-30 mortality risk was quantified by odds ratios (OR; with their associated 95% confidence Interval, 95% CI), the objective of the study being to take decisions for patients at a fixed-delay horizon. Factors with a p-value <0.20 in univariate were included in the multivariate analyses (logistic regression). A backward approach was used to simplify the model. The overlap between the different categorized factors was analysed using a Venn diagram. During the multivariate analyses, the collinearity between factors was analysed using variance inflation factors (VIF); with a threshold >1.5. The ability of the last model of 30-day mortality prediction was quantified by the area under the receiver operating characteristic (ROC) curve (AUC). A 5-fold cross-validation of the AUC of the model was performed to assess the optimism of this model.

No imputation of missing variables was performed. P-values <0.05 were considered significant. Analyses were performed using R software, version 3.3.2 (R: A language and environment for statistical computing. R Foundation for Statistical Computing, Vienna, Austria. URL http://www.R-project.org/).

**Table 1:** Patient characteristics of the total population and those who died at 30 days.

	Total population (n=231)	Day-30 mortality (n=60)	Variable	Total population (n=231)	Day-30 mortality (n=60)
Age (n=231)			ADL score (n=229)		
Mean (SD)	73.1 (7.4)	77.5 (7.3)	Mean (SD)	5.7 (0.7)	5.5 (0.9)
Range	60.1 - 91.4	62.0 - 91.4	Range	1.0 - 6.0	2.5 - 6.0
[60-70]; n (%)	80 (34.6%)	12 (15.0%)	6; n (%)	184 (80.3%)	35 (19.0%)
[70-80]; n (%)	107 (46.3%)	22 (20.6%)	<6; n (%)	45 (19.7%)	24 (53.3%)
>80; n (%)	44 (19.0%)	26 (59.1%)	IADL8 score (n=228)		
Sex (n=231); n (%)			Mean (SD)	6.8 (2.3)	5.4 (2.6)
male	174 (75.3%)	47 (27.0%)	Range	0.00 - 8.00	0.0 - 8.0
female	57 (24.7%)	13 (22.8%)	8; n (%)	154 (67.5%)	21 (13.6%)
Centre (n=231); n (%)			<8; n (%)	74 (32.5%)	37 (50.0%)
1	56 (24.2%)	12 (21.4%)	IADL4 score (n=228)		
2	71 (30.7%)	27 (38.0%)	Mean (SD)	3.4 (0.9)	3.2 (1.2)
3	38 (16.5%)	9 (23.7%)	Range	0.0 - 4.0	0.0 - 4.0
4	15 (6.5%)	0 (0.0%)	4; n (%)	177 (77.6%)	24 (19.2%)
5	27 (11.7%)	3 (11.1%)	<4; n (%)	51 (22.4%)	21 (47.1%)
6	10 (4.3%)	3 (30.0%)	Fried score (n=228)		
7	14 (6.1%)	6 (42.9%)	Mean (SD)	1.0 (1.4)	1.6 (1.6)
CIRS-G grade ≥2 number (n=231)			Range	0.0 - 5.0	0.0 - 5.0
Mean (SD)	2.0 (1.7)	2.6 (2.0)	≤2; n (%)	194 (85.1%)	40 (20.6%)
Range	0.0 - 8.0	0.0 - 7.0	≥3 (frail); n (%)	34 (14.9%)	18 (52.9%)
≤2; n (%)	152 (65.8%)	30 (19.7%)	Clinical Frailty Scale (n=219)		
>2; n (%)	79 (34.2%)	30 (38.0%)	Mean (SD)	2.5 (1.5)	3.3 (1.5)
Fall in the previous 6 months (n=231); n (%)			Range	1.0 - 8.0	1.0 - 7.0
No	201 (87.0%)	45 (22.4%)	<5; n (%)	195 (89.0%)	43 (22.1%)
Yes	30 (13.0%)	15 (50.0%)	≥5; n (%)	24 (11.0%)	14 (58.3%)

Data are n (%) unless otherwise stated. ADL: activities of daily living; IADL: instrumental ADL (IADL4 in 4 items; IADL8 in 8 items); CIRS-G: Cumulative Illness Rating scale-Geriatrics; CFS: Clinical Frailty Scale; SD: standard deviation.

**Table 2:** Risk factors of day-30-day mortality: univariate analyses.

Variable	OR [95% CI]	P-value
Age (per 10 years increase)	3.43 [2.13-5.53]	<0.001
Male	1.25 [0.62-2.53]	0.526
Grade ≥2 CIRS-G comorbidities	2.49 [1.36-4.56]	0.003
ADL score <6	4.86 [2.43-9.72]	<0.001
IADL8 score <8	6.33 [3.31-12.10]	<0.001
Fried score >2	4.33 [2.03-9.24]	<0.001
CFS ≥5 (Mildly frail)	4.18 [1.75-7.63]	0.001
Fall in the previous 6 months	3.46 [1.58-7.63]	0.002

ADL: activities of daily living; IADL: instrumental ADL; CIRS-G: Cumulative Illness Rating scale-Geriatrics; CFS: Clinical Frailty Scale

## RESULTS

Between March 7 and May 7, 290 patients were screened among whom 3 were admitted to >1 ICU and therefore 287 individual patients were identified; 40 were not included for lack of availability of the teams, and 16 were excluded because of patient or next of kin refusal. A total of 231 patients were included (Supplementary Fig. 1). There was no significant difference in the age, sex, and 30-day mortality between those included to those not included for reason of team availability.

**Figure 1.**
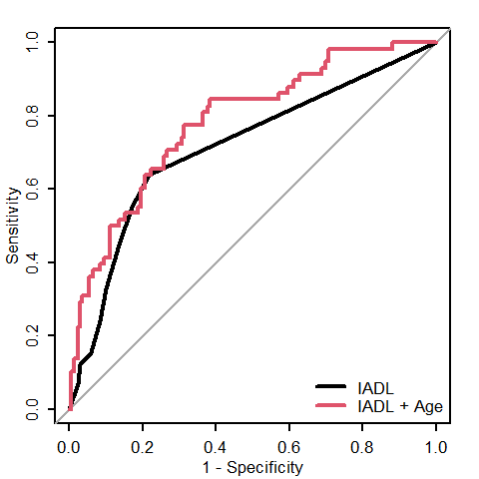
ROC curves.

Among those included, 209 (90.5%) patients were diagnosed via laboratory tests, and 22 (9.5%) via CT-scan diagnosis only. Most patients lived at home without formal care (80.9%, 182/225), 16.5% needed formal care services (35/225), and 3.6% lived in nursing homes (8/225). The median age was 73 years (range 60-91); 167 (75.0%) were men, 45 (19.7%) had an impairment in ADL and 74 (32.5%) in IADL8, 34 (15.2%) were frail according to Fried criteria and 24 (11.0%) according to CFS (Table 1). At admission, the median IGSII/SASP2 score was 39 (range 18-93), median PAO2/FiO2 ratio was 120 (range 49-532) and the median interval between the first COVID-19 symptoms and ICU admission was 9 days (range 0-41).

At day 30, 60 (26.0%) patients had died; the proportion ranged from 0 to 42.9% among the 7 ICUs (Table 1).

### Thirty-day mortality prediction model

In univariate analysis, the OR of death for each additional decade was 3.43 (95% CI [2.13; 5.53], p<0.001). The day-30 mortality rate in patients aged <70 years was 15%, and that in those aged ≥70 years was 31.8%. Comorbidities (grade ≥2 CIRS-G) were significantly associated with death at 30-days (OR: 2.48, 95% CI [1.36; 4.56]), as were all geriatric factors tested; the point estimates of which (OR) ranged from 3.46 (6 months fall history) to 6.33 (IADL8 score <8) (Table 2). There was a high overlap between IADL8 <8, ADL <6, Fried score >2, and CFS ≥5 on Venn diagram (Supplementary Fig. 2). The different multivariate models built are presented in Table 3. In the initial model including all variables significant in univariate analysis, the VIF associated with ADL, IADL, Fried scores, and CFS suggested collinearity between these factors. The final model included age (OR for an increase of 10 years: 2.94 [95% CI: 1.78; 5.04] and IADL8 <8 (OR:4.93, [95% CI: 2.52; 9.87]; Table 3). No significant interaction was found between age and IADL8 (p=0.827). The results of these analyses were independent from the thresholds chosen for the different studied factors; in fact, considering continuous factors (instead of categorized factors) led to the same final model (Supplementary Table 3).”

### Building of a triage tool for rapid evaluation of the risk/benefit balance of ICU admission

The logistic model including the age and IADL8 score as continuous variables led to an AUC of 0.78 [95% CI: 0.72, 0.85] (Fig. 1), significantly different from the AUC of age alone (AUC=0.71, [95% CI: 0.64, 0.79], p=0.049), and of IADL8 alone (AUC=0.72, [95% CI: 0.64, 0.79], p=0.027). The addition of either of frailty scores did not statistically improve the model. For comparative purposes, the AUC of ADL alone was 0.64 [95% CI: 0.57, 0.71], those of frailty according to Fried score and CFS were 0.66 [95% CI: 0.58, 0.74] and 0.72 [95% CI: 0.65, 0.79] respectively. The 5-fold cross validation for the model including age and IADL8 lead to a mean AUC of 0.80 [95% CI: 0.66 - 0.88]. According to simplified age categories by decades and binary IADL8 score (8 versus <8), 30-day risk of death ranged from 8.2% to 82.6% (Table 4), leading to three categories of mortality risk: low in patients aged <80 years with no IADL impairment (11.2% [95% CI : 6.4%-17.8%]) representing 58.8% of the population, intermediary in patients aged ≥80 years with no IADL impairment or aged <80 years with IADL impairment (33.8% [95% CI: 23.0%-46.0%]) representing 31.1% of the cohort, and high in patients aged ≥80 years with ≥1 IADL impairment (82.6% [95% CI: 61.2% - 95.0%]) representing 10.1% of the included population.

**Table 3:** Risk factors of day-30 mortality: multivariate analyses.

Model 1 (step 1)				Model 2 (step 2)				Model 3 (step 3)			
Variables	OR [95% CI]	p	VIF	Variables	OR [95% CI]	p	VIF	Variables	OR [95% CI]	p	VIF
Age (per 10-year increase)	3.26 [1.93-5.76]	<0.001	1.01	Age (per 10-year increase)	3.26 [1.93-5.76]	<0.001	1.01	Age (per 10-year increase)	3.27 [1.93-5.78]	<0.001	1.01
Grade ≥2 CIRS-G comorb. >2	1.45 [0.67-3.06]	0.34	1.20	Grade ≥2 CIRS-G comorb. >2	1.45 [0.67-3.05]	0.34	1.19	Grade ≥2 CIRS-G comorb. >2	1.47 [0.69-3.09]	0.32	1.17
ADL <6	1.13 [0.32-3.88]	0.85	2.57	IADL8 <8	3.05 [1.31-8.46]	0.003	1.45	IADL8 <8	3.26 [1.42-7.49]	0.006	1.41
IADL8 <8	2.93 [1.13-7.50]	0.028	1.79	Fried score >2	1.63 [0.52-5.05]	0.40	1.77	CFS ≥5	1.06 [0.35-3.17]	0.92	1.38
Fried score >2	1.60 [0.50-5.04]	0.43	1.82	CFS ≥5	0.84 [0.24-2.84]	0.77	1.72	Fall in last 6 mo YES	1.41 [0.53-3.70]	0.49	1.19
CFS ≥5	0.80 [0.21-2.94]	0.74	1.95	Fall in last 6 mo YES	1.28 [0.46-3.46]	0.64	1.26				
Fall in last 6 mo YES	1.24 [0.42-3.52]	0.69	1.40								
Model 4 (Step 4)				Model 5 (Step 5)				Model 6 (Final)			
Age (per 10-year increase)	2.84 [1.71-4.89]	<0.001	1.01	Age (per 10 years increase)	2.88 [1.74-4.95]	<0.001	1.01	Age (per 10-year increase)	2.94 [1.78-5.04]	<0.001	1.00
Grade ≥2 CIRS-G comorb. >2	1.41 [0.67-2.90]	0.36	1.13	IADL8 <8	5.17 [2.53-10.84]	<0.001	1.12	IADL8 <8	4.93 [2.52-9.87]	<0.001	1.00
IADL8 <8	4.12 [1.96-8.80]	<0.001	1.21	Fall in last 6 mo YES	1.39 [0.54-3.51]	0.49	1.13				
Fall in last 6 mo YES	1.33 [0.52-3.37]	0.55	1.14								

ADL: activities of daily living; IADL: instrumental ADL; CIRS-G: Cumulative Illness Rating scale-Geriatrics; CFS: Clinical Frailty Scale; OR: odd ratio; VIF: variance inflation factor

## DISCUSSION

In the present study age and IADL8 provided the best multivariate prediction model of 30-day mortality, with a good discriminative ability. These are highly objective parameters and poorly sensitive to inter-rater variation and provide an easy-to-use tool.

Accordingly, patients aged <80 years with no IADL impairment may benefit from ICU stay, and whether it is fair to admit COVID-19 patients aged ≥80 years with ≥1 IADL impairment is questionable. Conversely, it is less straight forward for the remaining patients. It is of note that the impact of functional impairment appears from 60 years of age; this may be considered as surprising since patients aged between 60 and 70 years are generally considered fit for ICU admission independently of any functional assessment, but are in line with previous reports that indicate that the first increase in mortality rate is found in patients aged ≥60 years [36]. For those aged ≥80 years without any IADL8 impairment, ICU admission is associated with the same benefit as younger patients with functional impairment and should therefore not be excluded based only on their age. Taken together, the results indicate that, in addition to age, IADL8 provides additional information to decide on ICU admission but there remains relative uncertainty for the patients of intermediary risk.

In such contexts and again according Christian et al, there is a need to provide a process and structure to mitigate chaos and improve the effectiveness of actions taken and triage criteria should be objective, ethical, transparent, applied equitably and publicly disclosed

Interestingly, IADL8 was significantly associated with 30-day in multivariate analysis while frailty scales were not. This absence of significant association is in relative contradiction with the literature highlighting the relationship between COVID-19 mortality and frailty [16, 17], and may be explained by several points. The most important is that the previously published study on the impact of CFS on ICU mortality included more than 6-fold more patients identified in a vast multicenter database [16], leading to a greater statistical power. In addition, as the present study included only those admitted to ICU, frail patients were poorly represented impairing *de facto* discrimination properties of frailty scores, and some discrepancies may lay between investigators when assessing the CFS [20, 21] leading to potential evaluation bias. However, these results are in line with previous data reported by Miles et al., who questioned the real impact of frailty facing COVID-19-infected patients, compared to non-COVID-19 patients [22]. Similarly, in their description of CFS in 1071 hospitalized older adults, Owen et al. demonstrated that COVID-19 infection was the main risk factor for death in the population, CFS appearing to make little incremental contribution to the hazard of dying in older people hospitalized with COVID-19 [23]. Another point to highlight is the absence of clear-cut discriminative threshold for CFS [16], as confirmed by the data presented herein, leading to question the arbitrary threshold of 5 proposed by the NICE [18] since the clinical distinction between CFS 4 and 5 may be considered as subjective [21]. The IADL8 score provided, in the present study, a good discriminative score, which is concordant with previous studies investigating ICU outcomes in older patients [37, 38]. For instance, according to Chelluri et al., the prehospitalization functional status (evaluated using IADL score) was significantly associated with short-term mortality, whereas age and comorbidities were associated with long-term mortality [37]. More recently, Giannotti et al. reported that, in the context of elective gastrointestinal oncogeriatric surgery, pre-morbid functional status (IADL) and cancer stage were the most significant predictors of one-year mortality, after having considered Frailty Index (FI) in the construction of the multivariate model [38]. Moreover, IADL integrates cognitive susceptibility, in addition to pure physical performance as demonstrated by Shimada et al. [39], and cognitive impairment is reported to be associated with short-term mortality (in-hospital death) after ICU admission [40].

**Table 4:** Day-30 mortality risk according to age categories and IADL8 score.

	Age categories		
	[60-70]	[70-80]	≥80
IADL8 score	n=78	n=107	n=43
<8 N=74	37.5% (6/16)) [15.2% - 64.6%]	34.3% (12/35) [19.1% - 52.2%]	82.6% (19/23) [61.2% - 95.0%]
8 N=154	8.2% (5/62) [2.7% - 17.8%]	13.9% (10/72) [6.9% - 24.1%]	30.0% (6/20) [11.9% - 54.3%]

IADL: instrumental activities of daily living

A strength of the present cohort study lays in the low rate of missing data in the *a posteriori* assessment of geriatric covariates of the patients 1 month before their admission to ICU. One of the limitations of this study may lie in the heterogeneity in the management of older patients during their stay in ICU; for instance it is widely documented that geriatric vulnerability may induce medical limitations during ICU stay, impacting mortality [41-43]. Another limitation is the change in the management of COVID-19 patients in ICU that have occurred since the first wave of the pandemic, and which may change the crude mortality risks in each risk category. In addition, differences in ICU admission criteria and care during ICU stay between the hospitals have also to be acknowledged.

To conclude, in addition to age, IADL8 provides information for the orientation of COVID-19 patients aged ≥60 years when discussing ICU admission. This tool could be of value as a non-admission decision help tool.

## Supplementary Materials

The Supplementary data can be found online at: www.aginganddisease.org/EN/10.14336/AD.2021.1004.


