# Supplementary material for: Senior-COVID-Rea Cohort Study: A Geriatric Prediction Model of 30-day Mortality in Patients Aged over 60 Years in ICU for Severe COVID-19
Source: Aging Dis. 2022 Apr 1;13(2):614–23. doi: 10.14336/AD.2021.1004 (PMC8947822; doi:10.14336/AD.2021.1004)
Supplement: Supplementary file 1 [file AD-13-2-614-s.pdf]

SUPPLEMENTARY DATA

Senior-COVID-Rea Cohort Study: A Geriatric Prediction Model of 30-day Mortality in Patients Aged over 60 Years in ICU for Severe COVID-19

Claire Falandry[1,2*], Laurent Bitker[3,4], Paul Abraham[5], Fabien Subtil[6,7], Vincent Collange[8], Baptiste Balança[9,10], Max Haïne[11], Céline Guichon[12,13], Christophe Leroy[14], Marie Simon[15], Amélie Malapert[16], Jean-Baptiste Pialat[17,18], Laurent Jallades[19], Alain Lepape[20,21], Arnaud Friggeri[20], Fabrice Thiolliere[20].

© 2021. Falandry C et al. Published online at http://www.aginganddisease.org/EN/10.14336/AD.2021.1004

SUPPLEMENTARY DATA

Supplementary Table 1. STROBE Statement. Checklist of items that should be included in reports of observational studies.

	Item No.	Recommendation	Page No.	Relevant text from manuscript
**Title and abstract**	1	(*a*) Indicate the study's design with a commonly used term in the title or the abstract	1	multicentre observational cohort study
		(*b*) Provide in the abstract an informative and balanced summary of what was done and what was found	4-5	
**Introduction**				
Background/rationale	2	Explain the scientific background and rationale for the investigation being reported	7	According to recent evidence from two studies, disease outcomes of COVID-19 patients admitted to hospital would be better predicted by frailty than either age or comorbidity.
Objectives	3	State specific objectives, including any prespecified hypotheses	8	we conducted a multicentre observational study to determine the clinical and biological covariates predictive of mortality in the population of patients over 60 years of age admitted in intensive care unit, with a specific attention paid to a retrospective and declarative assessment of their geriatric parameters 1 month before COVID-19 infection. This first analysis explores the respective impacts of various geriatric parameters and discusses their respective properties.
**Methods**				
Study design	4	Present key elements of study design early in the paper	9	Senior-COVID-Rea study is a multicenter observational cohort study
Setting	5	Describe the setting, locations, and relevant dates, including periods of recruitment, exposure, follow-up, and data collection	9	Data were collected across seven ICUs in Auvergne Rhone Alpes Region, France. A standardised case report format was used for recording data collected.
Participants	6	(*a*) *Cohort study*—Give the eligibility criteria, and the sources and methods of selection of participants. Describe methods of follow-up<br><br>*Case-control study*—Give the eligibility criteria, and the sources and methods of case ascertainment and control selection. Give the rationale for the choice of cases and controls<br><br>*Cross-sectional study*—Give the eligibility criteria, and the sources and methods of selection of participants	9	All patients aged 60 or older admitted to the participating ICUs with a diagnosis of COVID-19 were screened and included provided their (or their relative's) agreement. Diagnostic criteria were laboratory-confirmed SARS-CoV-2-positive swabs or a radiological diagnosis made by lung CT-scan consistent with COVID-19. Patients were excluded during data analysis only when duplicates were found, due to patients transferred from one ICU to the other from the participating centres. No other exclusion criteria were applied.

© 2021. Falandry C et al. Published online at http://www.aginganddisease.org/EN/10.14336/AD.2021.1004

		(*b*) *Cohort study*—For matched studies, give matching criteria and number of exposed and unexposed<br><br>*Case-control study*—For matched studies, give matching criteria and the number of controls per case	nc	
Variables	7	Clearly define all outcomes, exposures, predictors, potential confounders, and effect modifiers. Give diagnostic criteria, if applicable	10	In this analysis, only primary outcome was analysed, ie the day-30 mortality (time from ICU admission).
Data sources/ measurement	8*	For each variable of interest, give sources of data and details of methods of assessment (measurement). Describe comparability of assessment methods if there is more than one group	10	In this analysis, only primary outcome was analysed, ie the day-30 mortality (time from ICU admission).
Bias	9	Describe any efforts to address potential sources of bias	11	Accrual bias:<br><br>Clinical teams at each site screened inpatient admission lists for eligibility. Screening logs of eligible participants were retained at each site.<br><br>Statistical bias:<br><br>The overlap between the different factors was analyzed through a Venn diagram, using the categorized version of the different factors. Moreover, during the multivariate analyses, the collinearity between factors was analyzed through variance inflation factors (VIF). The ability of the last model obtained to identified patients that died during the 30 days following ICU admission was quantified by the area under the ROC curve (AUC); it was compared to the AUC of age alone. A 5-fold cross-validation of the AUC of the model obtained was performed to assess the optimism of this model.<br><br>No imputation of missing variables was performed. P-values less than 0.05 were considered significant.
Study size	10	Explain how the study size was arrived at	10	The first hypothesis of Senior-COVID-Rea was based of the first results of first Chinese retrospective results (1): considering a single analysis variable (age), with expected mortality of 30% in patients under 70 years of age, and 70% in patients over 70 years of age (with 40% of patients over 70 years of age), a total of 130 patients was expected to show a statistically significant difference between these two groups with a power of 90% (bilateral alpha risk test of 5%). Since the analysis considered the integration of several factors, considering 15 factors, hoping for a coefficient of determination of 0.5 of the model, to achieve an optimism of less than

© 2021. Falandry C et al. Published online at http://www.aginganddisease.org/EN/10.14336/AD.2021.1004

				10%, 185 patients were to be included (criterion 1 of Riley, Snell et al, (15)).<br><br>After the publication of data on mortality in ICU in Lombardy region, Italy in April 2020 (2), considering that a stopping of the trial at 185 patients would impair its statistical power and induce a potential risk of patients' selection bias, the scientific committee decided, on the 7th May, that all the patients admitted to ICU before that date - that corresponded to the end of the first COVID wave – should be screened and proposed the study without any patients' number limitation.<br><br>This sample size calculation was modified on Clinicaltrials.gov site accordingly (July 28, 2020).
Quantitative variables	11	Explain how quantitative variables were handled in the analyses. If applicable, describe which groupings were chosen and why	10	Continuous variables were described by the mean, standard deviation (SD), and range. Categorized variables were described by the frequency and percentage of each modality. Common thresholds were applied: CFS ≥5, ADL <6, IADL8 <8, Fried score >2.
Statistical methods	12	(*a*) Describe all statistical methods, including those used to control for confounding	10	The effect of factors on day-30 mortality risk was quantified by odds ratios (with their associated 95% Confidence Interval, 95% CI). Factors with a p-value less than 0.20 is univariate were included in the multivariate analyses (logistic regression)
		(*b*) Describe any methods used to examine subgroups and interactions	10	The overlap between the different categorized factors was analyzed through a Venn diagram. During the multivariate analyses, the collinearity between factors was analyzed through variance inflation factors (VIF); with a threshold above 1.5.
		(*c*) Explain how missing data were addressed	11	No imputation of missing variables was performed
		(*d*) *Cohort study*—If applicable, explain how loss to follow-up was addressed<br><br>*Case-control study*—If applicable, explain how matching of cases and controls was addressed<br><br>*Cross-sectional study*—If applicable, describe analytical methods taking account of sampling strategy	na	Not applicable: no patient loss of follow up for the primary endpoint
		(*e*) Describe any sensitivity analyses	11	Subgroup analyses are not relevant in this study, but a cross-validation analysis was performed to assess the optimism of the model.
**Results**				
Participants	13*	(a) Report numbers of individuals at each stage of study—eg numbers potentially eligible, examined for eligibility, confirmed eligible,	11	STROBE flow diagram (Figure 1)

© 2021. Falandry C et al. Published online at http://www.aginganddisease.org/EN/10.14336/AD.2021.1004

		included in the study, completing follow-up, and analysed		
		(b) Give reasons for non-participation at each stage	11	STROBE flow diagram (Figure 1)
		(c) Consider use of a flow diagram	11	STROBE flow diagram (Figure 1)
Descriptive data	14*	(a) Give characteristics of study participants (eg demographic, clinical, social) and information on exposures and potential confounders	11	Table 1
		(b) Indicate number of participants with missing data for each variable of interest	11	Table 1
		(c) *Cohort study*—Summarise follow-up time (eg, average and total amount)	na	Short time endpoint (30 days), no missing data
Outcome data	15*	*Cohort study*—Report numbers of outcome events or summary measures over time	11	Table 2
		Case-control study—Report numbers in each exposure category, or summary measures of exposure		
		Cross-sectional study—Report numbers of outcome events or summary measures		
Main results	16	(*a*) Give unadjusted estimates and, if applicable, confounder-adjusted estimates and their precision (eg, 95% confidence interval). Make clear which confounders were adjusted for and why they were included	11	Table 3
		(*b*) Report category boundaries when continuous variables were categorized	11	Table 3
		(*c*) If relevant, consider translating estimates of relative risk into absolute risk for a meaningful time period	na	
Other analyses	17	Report other analyses done—eg analyses of subgroups and interactions, and sensitivity analyses	10, 12	The overlap between the different factors was analyzed through a Venn diagram, using the categorized version of the different factors. Moreover, during the multivariate analyses, the collinearity between factors was analyzed through variance inflation factors (VIF).<br>Table 4
**Discussion**				
Key results	18	Summarise key results with reference to study objectives	12	Our results confirm our primary hypothesis, patients over 75 having, in this study, an almost 5-fold higher risk of D30 mortality, compared to younger ones (OR 4.82 (95%CI, 2.56-9.06), p<0.001). More precisely, D30 death rates varied from 15% in patients aged

© 2021. Falandry C et al. Published online at http://www.aginganddisease.org/EN/10.14336/AD.2021.1004

				60 to 69, 21% in patients aged 70 to 79, to 59% in patients over 80.
Limitations	19	Discuss limitations of the study, taking into account sources of potential bias or imprecision. Discuss both direction and magnitude of any potential bias	14	due to the study structure evaluating patients admitted to ICU, frail patients were poorly represented in the cohort, impairing de facto discrimination properties of frailty scores.
Interpretation	20	Give a cautious overall interpretation of results considering objectives, limitations, multiplicity of analyses, results from similar studies, and other relevant evidence	15	When considering functional impairment, one may be surprised since impairment in IADL appears more discriminative than impairment in ADL, as ADLs are usually considered as lately affected in the spectrum of dependence. Moreover, in their recently published model, Bousquet et al identified impairment in ADL but not in IADL4 as significantly associated with D30 mortality in an older COVID-19 population admitted in geriatric wards (26). One response may be linked to a low statistical power, since IADL impairment concerned 33% of the population when ADL impairment affected “only” 20% of the population, ADL impaired patients being frequently considered as unfit for resuscitation.
Generalisability	21	Discuss the generalisability (external validity) of the study results	16	To conclude, age and IADL provide independent prognostic factors for D30 mortality in patients over 60 admitted in ICU for severe COVID-19 infection. Our triage model may be considered as useful to integrate into the individualized resuscitation proposal whereas exponential increase in COVID-19 incidence induces increasing constraints on healthcare systems. A future study is about to be launched, to externally validate the model.
**Other information**				
Funding	22	Give the source of funding and the role of the funders for the present study and, if applicable, for the original study on which the present article is based	16	All the authors declare a grant from Hospices Civils de Lyon, during the conduct of the study, for Clinical Research Assistants and statistical analysis, no other competing interests with the considered topic. The funder of the study had no role in study design, data collection, data analysis, data interpretation, or writing of the report.

*Give information separately for cases and controls in case-control studies and, if applicable, for exposed and unexposed groups in cohort and cross-sectional studies.

Note: An Explanation and Elaboration article discusses each checklist item and gives methodological background and published examples of transparent reporting. The STROBE checklist is best used in conjunction with this article (freely available on the Web sites of PLoS Medicine at http://www.plosmedicine.org/, Annals of Internal Medicine at http://www.annals.org/, and Epidemiology at http://www.epidem.com/). Information on the STROBE Initiative is available at www.strobe-statement.org.

SUPPLEMENTARY DATA

Supplementary Table 2. TRIPOD checklist.

Section/Topic	Item	Checklist Item	Page
**Title and abstract**			
Title	1	Identify the study as developing and/or validating a multivariable prediction model, the target population, and the outcome to be predicted.	1
Abstract	2	Provide a summary of objectives, study design, setting, participants, sample size, predictors, outcome, statistical analysis, results, and conclusions.	4
**Introduction**			
Background and objectives	3a	Explain the medical context (including whether diagnostic or prognostic) and rationale for developing or validating the multivariable prediction model, including references to existing models.	7-8
	3b	Specify the objectives, including whether the study describes the development or validation of the model or both.	8
**Methods**			
Source of data	4a	Describe the study design or source of data (e.g., randomized trial, cohort, or registry data), separately for the development and validation data sets, if applicable.	9-10
	4b	Specify the key study dates, including start of accrual; end of accrual; and, if applicable, end of follow-up.	11
Participants	5a	Specify key elements of the study setting (e.g., primary care, secondary care, general population) including number and location of centres.	9, 17
	5b	Describe eligibility criteria for participants.	9
	5c	Give details of treatments received, if relevant.	na
Outcome	6a	Clearly define the outcome that is predicted by the prediction model, including how and when assessed.	
	6b	Report any actions to blind assessment of the outcome to be predicted.	na
Predictors	7a	Clearly define all predictors used in developing or validating the multivariable prediction model, including how and when they were measured.	10
	7b	Report any actions to blind assessment of predictors for the outcome and other predictors.	na
Sample size	8	Explain how the study size was arrived at.	10
Missing data	9	Describe how missing data were handled (e.g., complete-case analysis, single imputation, multiple imputation) with details of any imputation method.	11
Statistical analysis methods	10a	Describe how predictors were handled in the analyses.	10, 11
	10b	Specify type of model, all model-building procedures (including any predictor selection), and method for internal validation.	10, 11
	10d	Specify all measures used to assess model performance and, if relevant, to compare multiple models.	10, 11
Risk groups	11	Provide details on how risk groups were created, if done.	10
**Results**			
Participants	13a	Describe the flow of participants through the study, including the number of participants with and without the outcome and, if applicable, a summary of the follow-up time. A diagram may be helpful.	11 (Figure 1)
	13b	Describe the characteristics of the participants (basic demographics, clinical features, available predictors), including the number of participants with missing data for predictors and outcome.	11 (Table 1)

© 2021. Falandry C et al. Published online at http://www.aginganddisease.org/EN/10.14336/AD.2021.1004

SUPPLEMENTARY DATA

Model development	14a	Specify the number of participants and outcome events in each analysis.	11 (table 3)
	14b	If done, report the unadjusted association between each candidate predictor and outcome.	12 (table 4)
Model specificatio[n]	15a	Present the full prediction model to allow predictions for individuals (i.e., all regression coefficients, and model intercept or baseline survival at a given time point).	12 (table 4 & 5, Figure 3)
	15b	Explain how to the use the prediction model.	12, 16
Model performanc[e]	16	Report performance measures (with CIs) for the prediction model.	12 (5, Figure 3)
**Discussion**			
Limitations	18	Discuss any limitations of the study (such as nonrepresentative sample, few events per predictor, missing data).	14-16
Interpretation	19b	Give an overall interpretation of the results, considering objectives, limitations, and results from similar studies, and other relevant evidence.	14-16
Implications	20	Discuss the potential clinical use of the model and implications for future research.	14-16
**Other information**			
Supplementary information	21	Provide information about the availability of supplementary resources, such as study protocol, Web calculator, and data sets.	17
Funding	22	Give the source of funding and the role of the funders for the present study.	16

Supplementary Table 3. Risk factors of day-30 mortality: multivariate analyses (continuous variables).

**Model 1 (step 1)**				**Model 2 (step 2)**				**Model 3 (step 3)**			
**Variables**	**OR [95%CI]**	**p**	**VIF**	**Variables**	**OR (95%CI]**	**p**	**VIF**	**Variables**	**OR (95%CI]**	**p**	**VIF**
Age (per 10 years increase)	3,41 [1,99, 6,11)	<0,001	1·08	Age (per 10 years increase)	2,96 [1,79, 5,09]	<0,001	1·03	Age (per 10 years increase)	3 [1,82, 5,16]	<0,001	1·03
Nb of grade≥2 CIRS-G comorb.	1,10 [0,89, 1,36]	0,395	1·19	Nb of grade≥2 CIRS-G comorb.	1,10 [0,09, 1,35]	0,356	1·14	Nb of grade≥2 CIRS-G comorb.	1,11 [0,9, 1,36]	0,326	1·14
ADL	1,12 [0,58, 2,31]	0,747	2·56	ADL	1,28 [0,69, 2,57]	0,451	2.35	IADL8	0,8 [0,68, 0,94]	0,008	1·37
IADL8	0,81 [0,63, 1,05]	0,113	3.19	IADL8	0,76 [0,62, 0,94]	0,011	2.20	Fried score	1,09 [0,82, 1,44]	0,552	1·45
Fried score	1,15 [0,85, 1,57]	0,362	1·63	Fried score	1,12 [0,83, 1,50]	0,454	1·56	Fall in last 6 mo.	1,28 [0,46, 3,4]	0,624	1·23
CFS	1,02 [0,69, 1,53]	0,908	3.33	Fall in last 6 mo.	1,36 [0,49, 3,69]	0,553	1·26				
Fall in last 6 mo.	1,38 [0,49, 3,78]	0,531	1.26								
**Model 4 (Step 4)**				**Model 5 (Step 5)**				**Model 6 (Final)**			
Age (per 10 years increase)	3,04 [1,84, 5,22]	<0,001	1·02	Age (per 10 years increase)	3,11 [1,89, 5,33]	<0,001	1·01	Age (per 10 years increase]	3,24 [1,98, 5,53]	<0,001	1.00
Nb of grade≥2 CIRS-G comorb.	1,11 [0,91, 1,36]	0,311	1·14	Nb of grade≥2 CIRS-G comorb.	1,13 [0,93, 1,38]	0,223	1·08	IADL8	0,75 [0,65, 0,86]	<0,001	1.00
IADL8	0,79 [0,68, 0,93]	0,005	1·32	IADL8	0,77 [0,67, 0,89]	<0,001	1·07				
Fried score	1,11 [0,84, 1,46]	0,458	1·37								

© 2021. Falandry C et al. Published online at http://www.aginganddisease.org/EN/10.14336/AD.2021.1004

SUPPLEMENTARY DATA

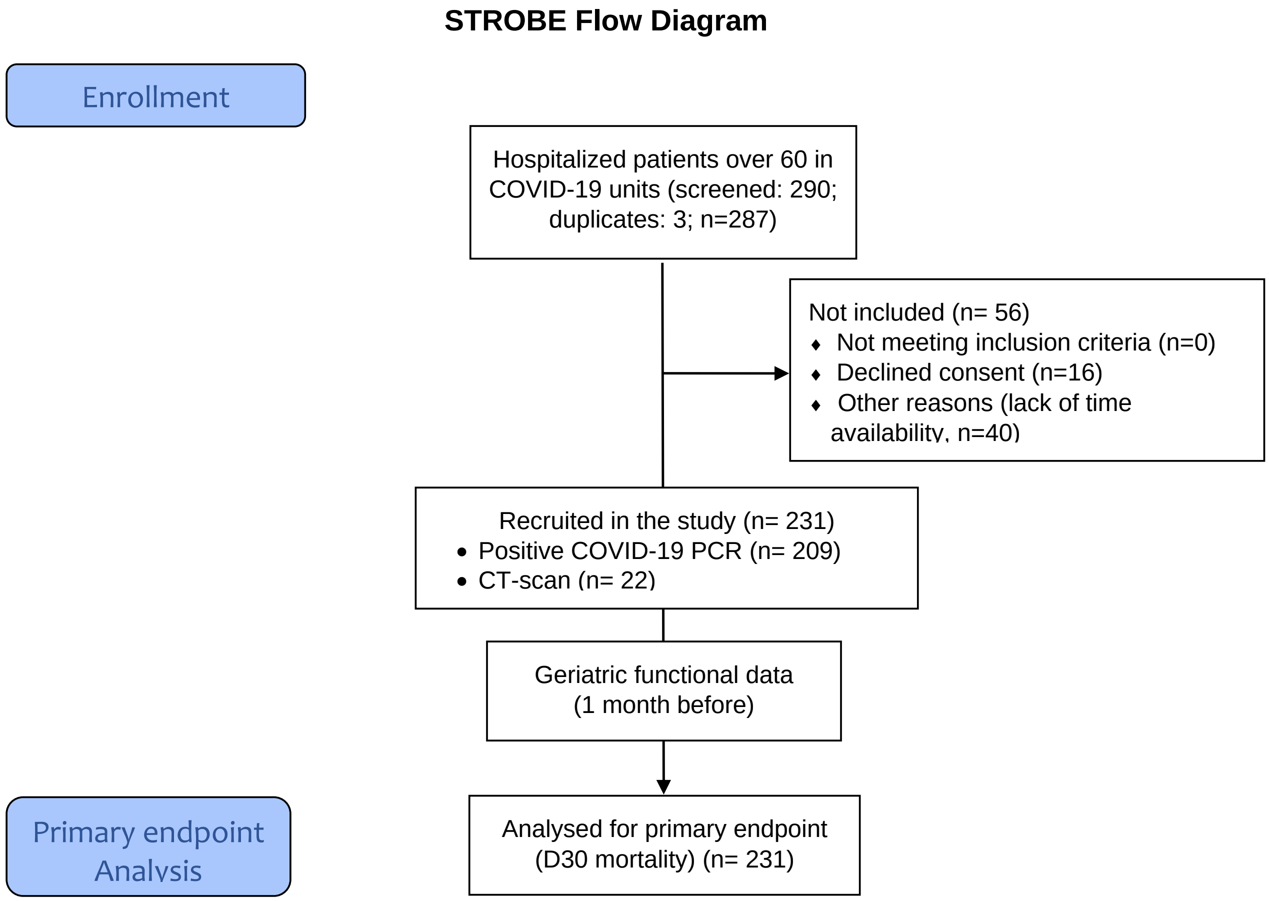

Supplementary Figure 1. STROBE Flow diagram

© 2021. Falandry C et al. Published online at http://www.aginganddisease.org/EN/10.14336/AD.2021.1004

SUPPLEMENTARY DATA

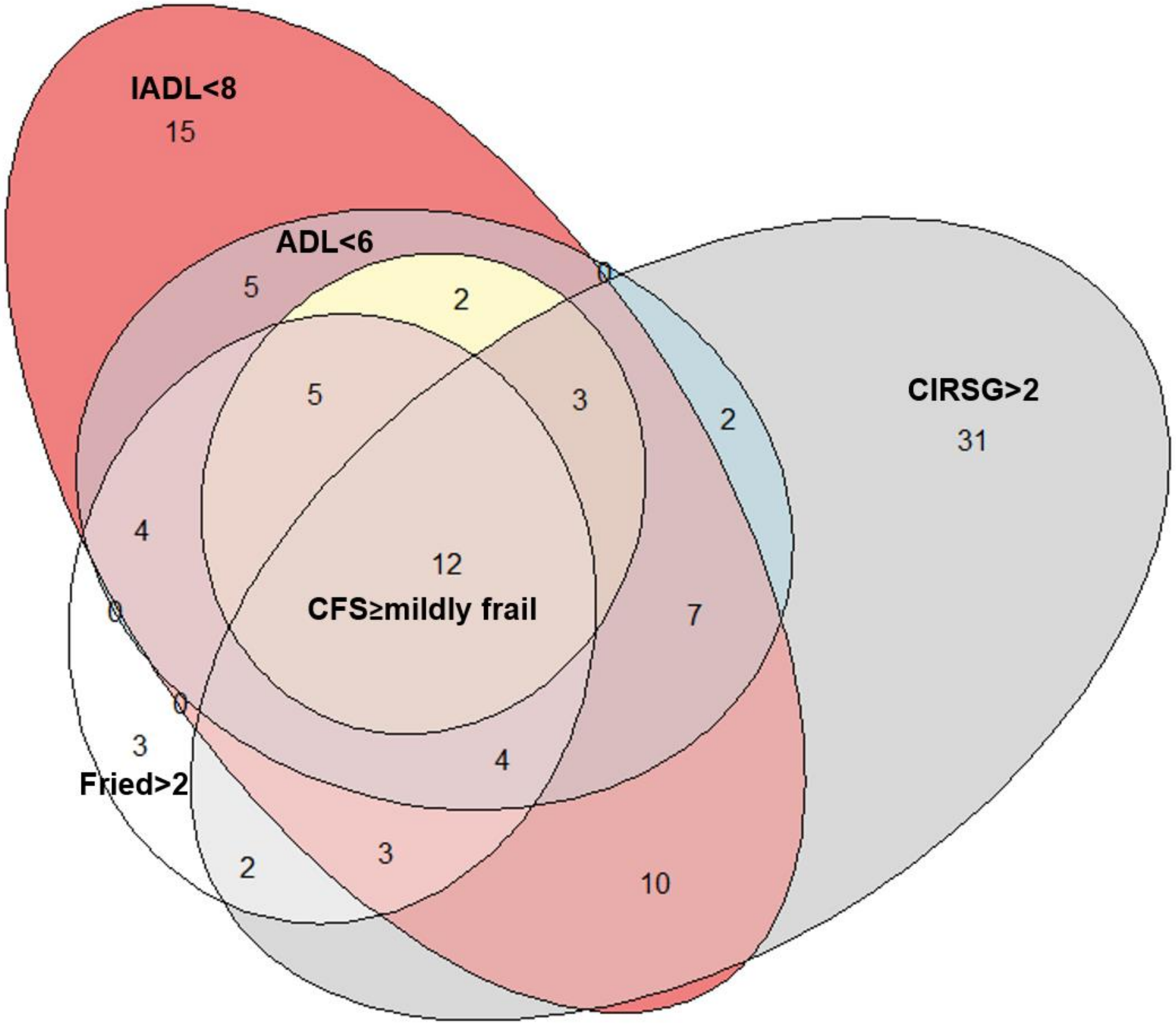

Supplementary Figure 2. Venn diagram displaying extent of overlap of geriatric vulnerability parameters in Senior-COVID-Rea population (n=231). ADL: patients with ≥1 impaired activity of daily living (n=45); CFS≥5 (n=24); CIRS-G: patients with 3 or more grade≥2 CIRS-G comorbidities (n=79); IADL: patients with ≥1 impaired activity of daily living according IADL8 score (n=74).

© 2021. Falandry C et al. Published online at http://www.aginganddisease.org/EN/10.14336/AD.2021.1004
